# Supplementary material for: Validation and Data-Integration of Yeast-Based Assays for Functional Classification of BRCA1 Missense Variants
Source: Int J Mol Sci. 2022 Apr 6;23(7):4049. doi: 10.3390/ijms23074049 (PMC8999655; doi:10.3390/ijms23074049)
Supplement: Supplementary file 1 [file ijms-23-04049-s001.zip › Figures S1 and S2.pdf]

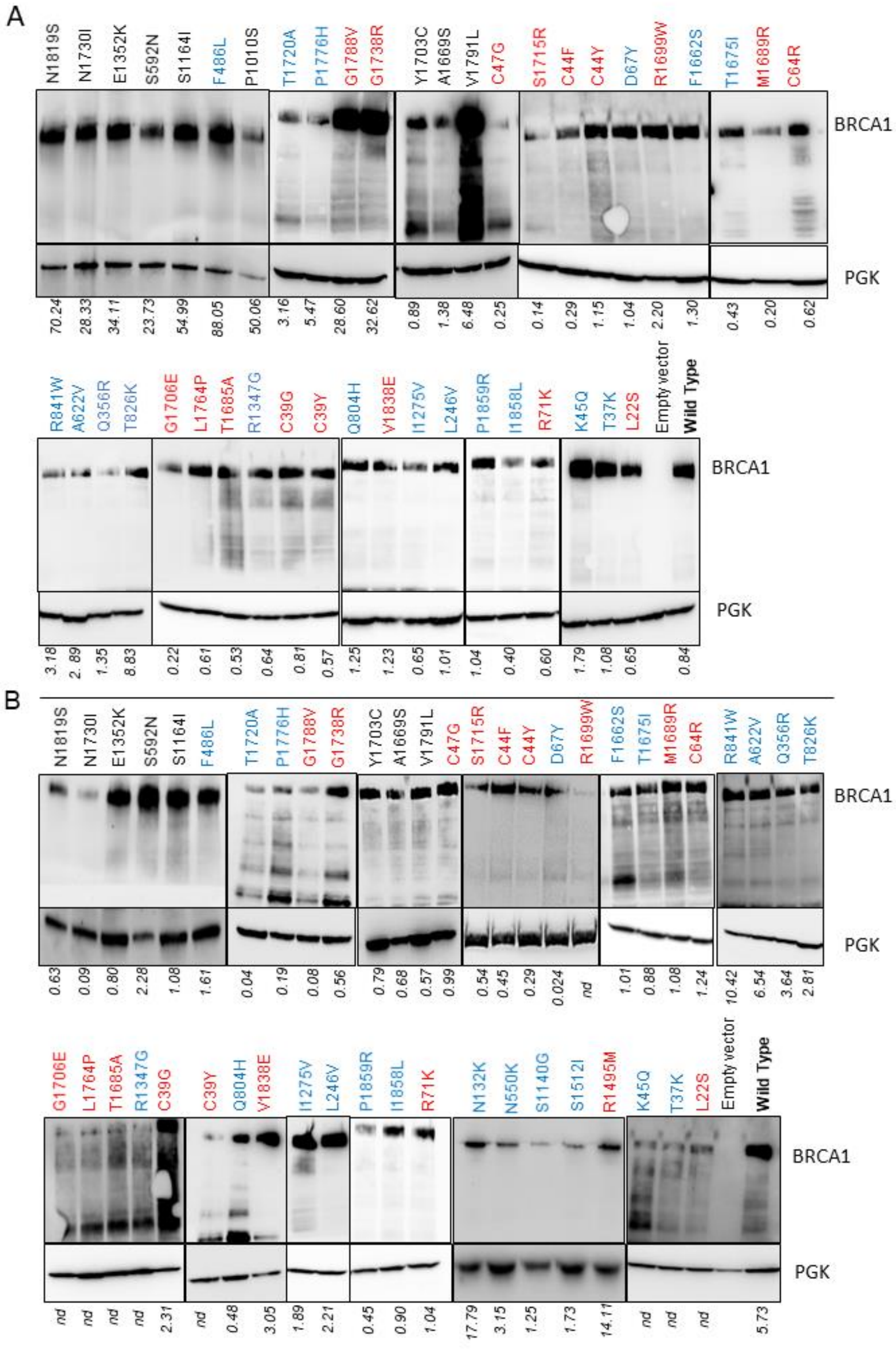

Figure S1. Yeast strains express BRCA1 missense variants. BRCA1 protein level was determined in yeast cell extracts from both RSY6 (A) and RS112 (B) strains transformed with BRCA1 expression vector after 24h induction in galactose medium, as described in Materials and Methods. BRCA1p is analysed using Anti-BRCA1 mouse antibody diluted 1:300 (clone MS110, Calbiochem, Italy). As loading control, we determined the level of 3-phosphoglycerate kinase (PGK) with the anti-αPGK antibody (Invitrogen, OR USA). Below the gels is reported BRCA1 band intensity normalized to band intensity of PGK (loading control). Western blot images were retrieved with ChemiDoc™ MP System (Biorad) and band intensity was determined by Image lab (Biorad). Pathogenic variants are in red, benign variants in blue and VUS are black. ND not determined.

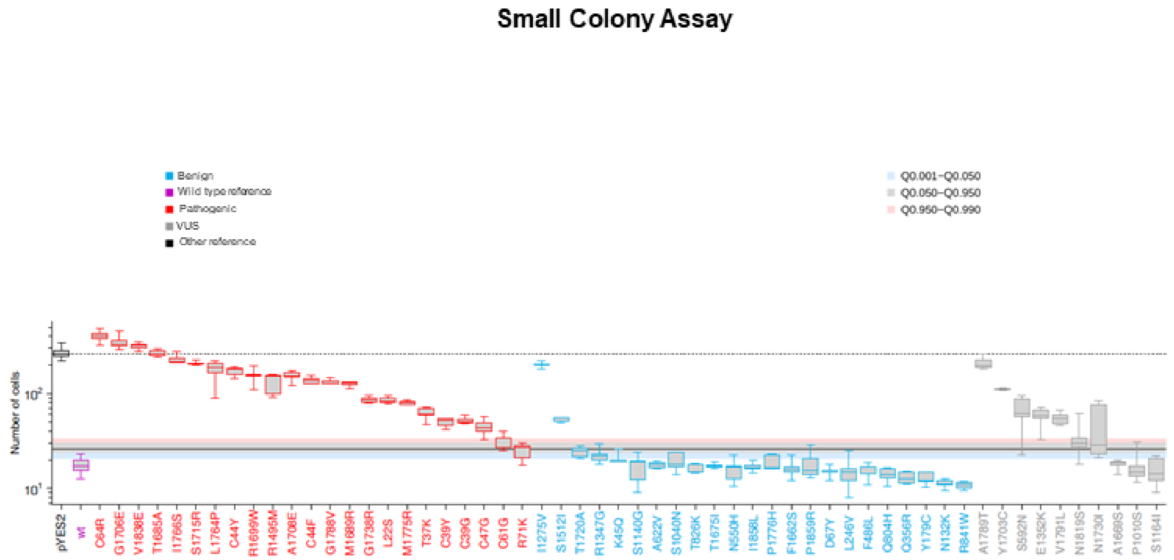

Figure S2. Waterfall distribution of data from SCP assay. Pathogenic and benign variants were also tested in SCP assay. Data are reported as box-and-whisker plot; red, blue and grey box indicate pathogenic, benign and VUS. Box central bar represents the median; box indicates the interquartile range (50% of data distribution), whiskers show the extreme values. Horizontal dotted line represents the median of control (empty pYES2). Thick line is the best cut-off; pink, grey and light blue areas define 4%, 90% and 4.9% of total distribution. These parameters are determined by the ProClass toolbox.
